# Usefulness of Ligamentotaxis With Balloon Kyphoplasty for Delayed Lumbar Radiculopathy Resulting From Osteoporotic Vertebral Fracture: A Case Report

**DOI:** 10.7759/cureus.76450

**Published:** 2024-12-26

**Authors:** Fumiaki Fujihara, Hisaaki Uchikado, Yukino Irie, Tooru Inoue, Hiroshi Abe

**Affiliations:** 1 Department of Neurosurgery, Hakujyuji Hospital, Fukuoka, JPN; 2 Department of Neurological Surgery, Uchikado Neuro-Spine Clinic, Fukuoka, JPN; 3 Department of Neurosurgery, Fukuoka University, Fukuoka, JPN

**Keywords:** balloon kyphoplasty, delayed neurological deficit, ligamentotaxis, osteoporotic vertebral fracture, radiculopathy

## Abstract

Osteoporotic vertebral fractures (OVF) commonly occur at the thoracolumbar junction, and delayed neurological deficits are rare. Here, a 76-year-old female presented with low back pain and the late onset of symptoms characterized by lumbar radiculopathy. She had been suffering from lower back pain for four weeks and developed lumbar radiculopathy in the left L3 area. The lumbar magnetic resonance (MR) and three-dimensional computed tomography (3D-CT) demonstrated an L3 OVF with bone fragments at the entrance of the neural foramen. She ultimately underwent a balloon kyphoplasty (BKP), as conservative treatment was unsuccessful for six weeks. The bone fragment had been reduced with ligamentotaxis caused by BKP, and the pain in the left L3 area disappeared immediately after the surgery. 3D-CT is established in addition to MR for the diagnosis of delayed radiculopathy by lumbar OVF. BKP alone using the ligamentotaxis of the posterior longitudinal ligament is a minimally invasive and rational surgical method.

## Introduction

Randomized trial studies have demonstrated that balloon kyphoplasty (BKP) is an effective and minimally invasive surgical treatment for managing osteoporotic vertebral fractures (OVFs) in elderly patients. BKP has been shown to provide acute pain relief and correct kyphosis, making it a valuable option for addressing these fractures [1,2]. Furthermore, reduction with BKP may improve neurological symptoms caused by vertebral collapse or compression from bone fragments. However, in cases of delayed neurological deficits associated with OVFs, BKP alone is typically not sufficient. In such situations, decompression combined with fusion surgery is often recommended to address the underlying pathology effectively [3].

In this report, a 76-year-old female with an L3 OVF presented with delayed L3 radiculopathy. Her symptoms, originating from the L3 radiculopathy, were resolved with BKP alone.

## Case presentation

**Figure 1:**
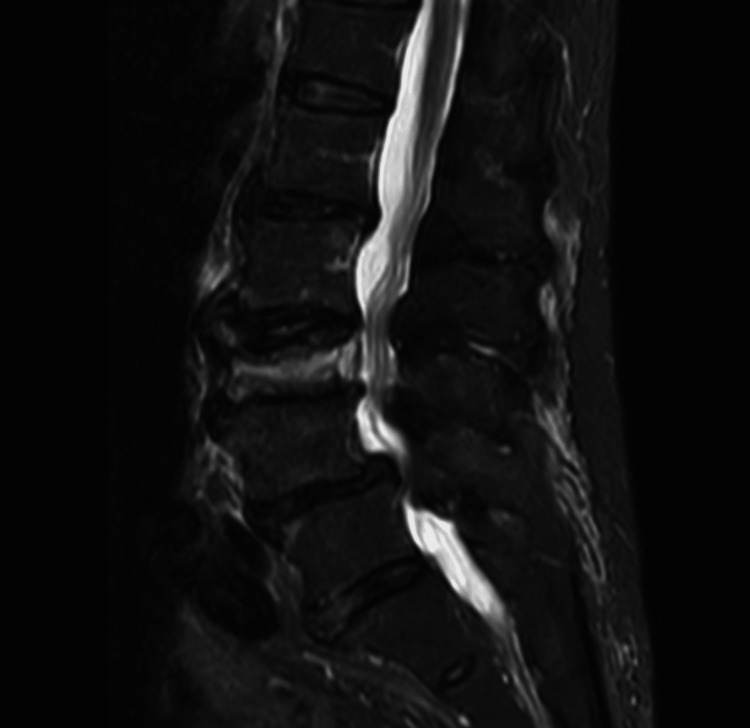
Sagittal view of lumbar MRI T2-STIR Magnetic resonance imaging (MRI T2-STIR) showed an L3 acute osteoporotic vertebral fracture (OVF). T2-STIR: T2-weighted short-tau inversion recovery

**Figure 2:**
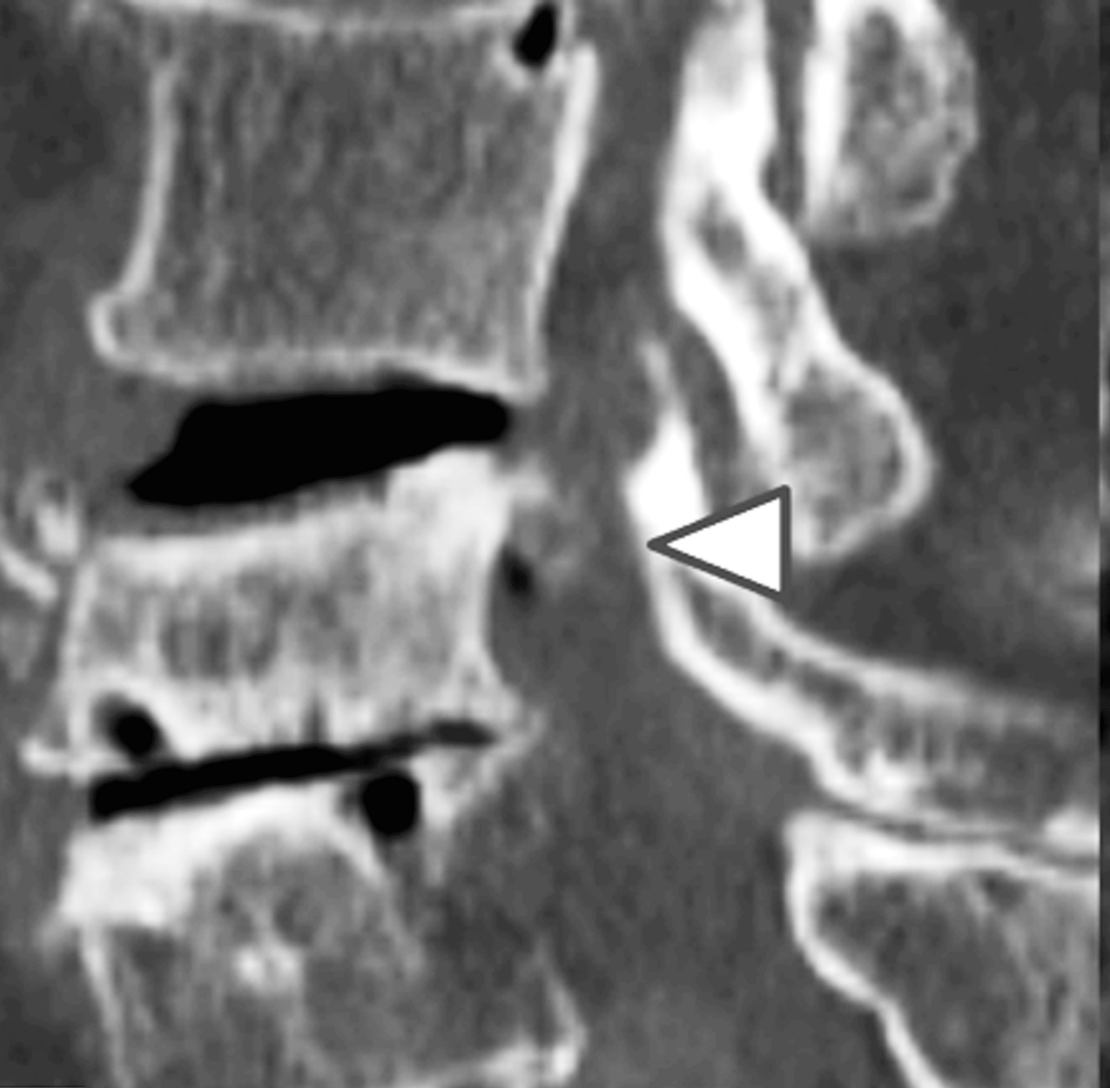
Sagittal view of lumbar 3D-CT Three-dimensional computed tomography (3D-CT) showed a retropulsed bone fragment (white arrowhead) with L3 OVF. OVF: osteoporotic vertebral fracture

A 76-year-old female presented with low back pain and severe leg pain and had been treated only with oral non-steroidal anti-inflammatory drugs, without receiving corset therapy. She has no history of internal medical diseases. The lumbar magnetic resonance (MR) demonstrated OVF at L3 (Figure 1); four weeks later, she developed severe left L3 lumbar radiculopathy with paralysis of the left iliopsoas and quadriceps. Manual muscle testing revealed muscle weakness with a score of 2 for the iliopsoas and 3 for the quadriceps, and the straight leg-raising test was negative. She experienced persistent severe pain and sensory disturbances in the left L3 region. Three-dimensional computed tomography (3D-CT) revealed a partial posterior wall fracture as an incomplete burst fracture, with a bone fragment protruding into the left-side entrance of the neural foramen (Figure 2). She received conservative treatment for six weeks, but her neurological symptoms did not improve.

We performed BKP with a left unilateral approach, expecting ligamentotaxis of the posterior longitudinal ligament (PLL) in a hybrid operating room (Philips Azurion 7 B20/15, Philips, Netherlands). The reduction of retropulsed bone fragments with ligamentotaxis could be confirmed using intraoperative cone beam 3D-CT. If adequate reduction was not achieved, additional posterior decompression was planned.

**Figure 3:**
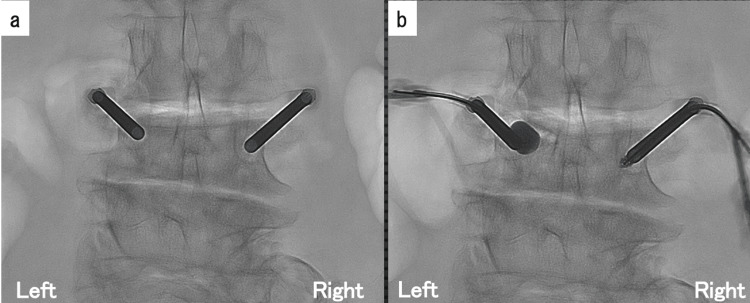
Balloon kyphoplasty (BKP) a) Before balloon kyphoplasty (BKP), X-ray showed the left side vertebral height loss; b) After BKP, X-ray revealed corrected vertebral body height.

**Figure 4:**
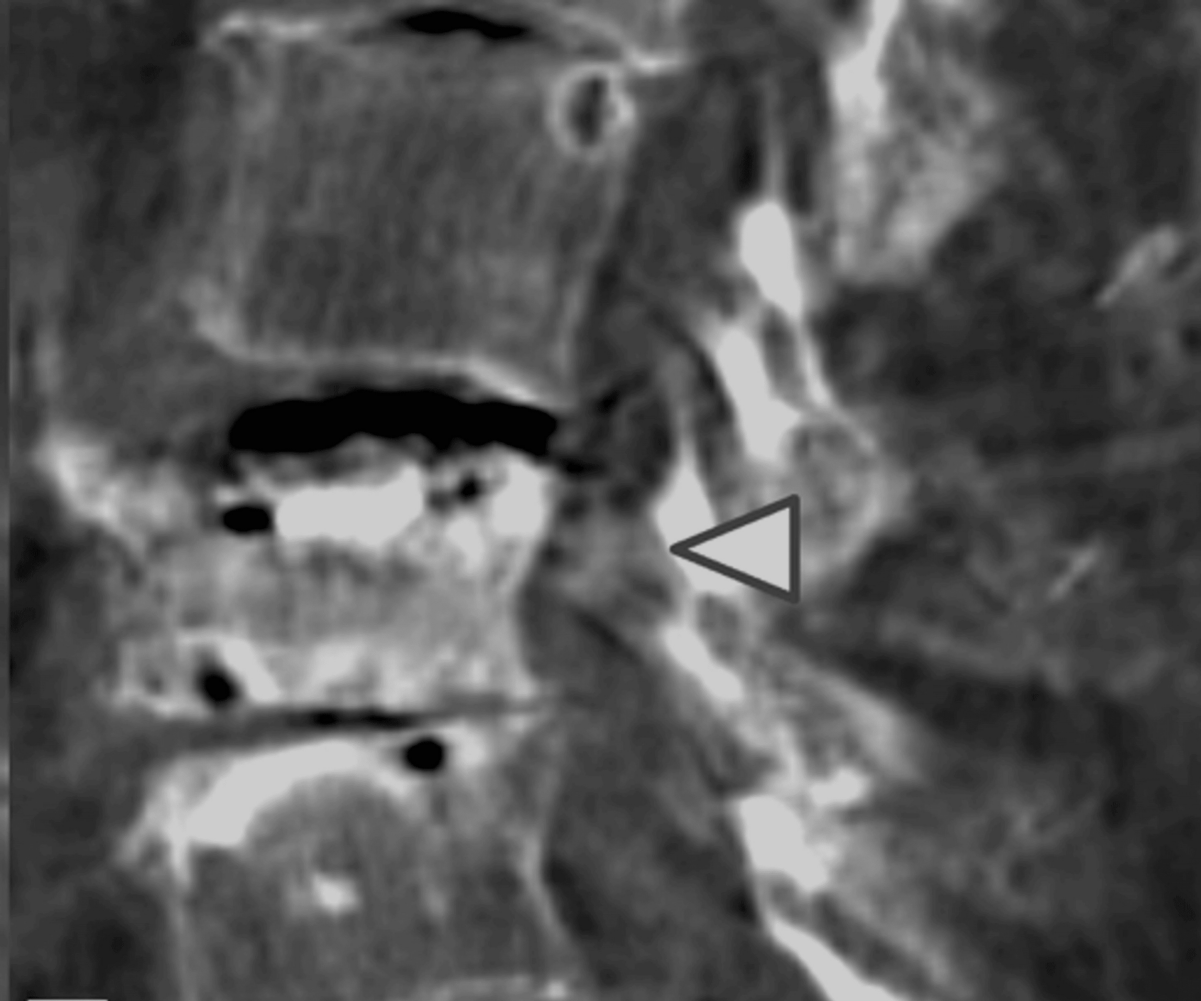
Sagittal view of intraoperative cone-beam 3D-CT Intraoperative cone-beam 3D-CT showed a reduction of the bone fragment (white arrowhead).

**Figure 5:**
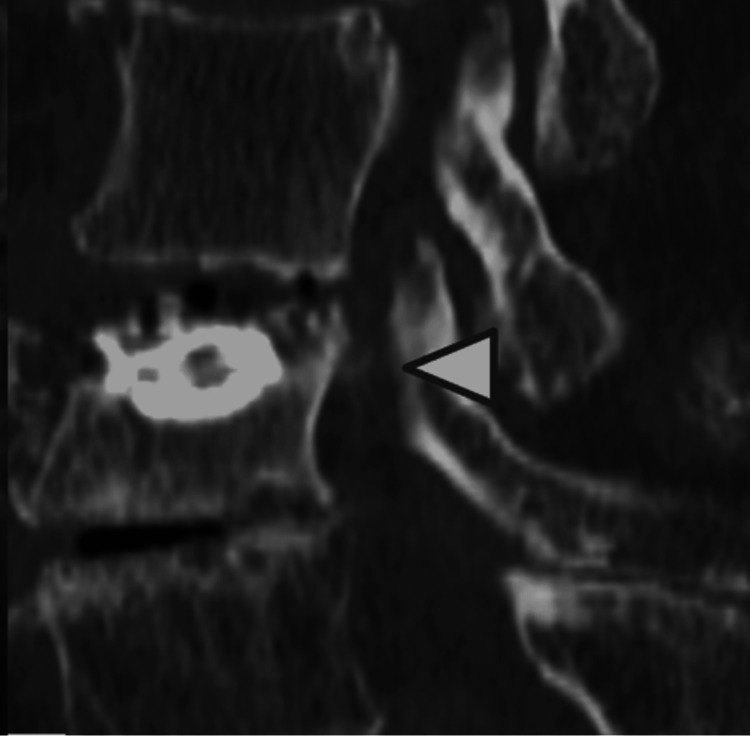
Sagittal view of postoperative 3D-CT Postoperative 3D-CT revealed a reduction of the bone fragment (white arrowhead).

The skin incision was made approximately 0.5 cm in length at 1 cm lateral to the center of the pedicle. Under fluoroscopic guidance, a trocar was inserted into the vertebral body, passing through the pedicle. Intraoperative X-ray showed correction of the left vertebral body height by a balloon inflated with a contrast-saline solution under a pressure of 20 atm (Figures 3a-3b). The cement was carefully injected using biplane fluoroscopy. The cone beam 3D-CT during BKP revealed that the retropulsed bone fragment had been reduced (Figure 4). Postoperatively, the neurological symptoms disappeared completely, and 3D-CT revealed a reduction of retropulsed bone fragments (Figure 5). At the three-month postoperative follow-up, the patient showed no recurrence of neurological symptoms. However, follow-up was discontinued due to dementia.

## Discussion

In the treatment of OVF with delayed neurological deficits, surgical techniques are divided into three types: anterior, posterior, and posteroanterior surgery. The posterior surgery could be further divided into posterior decompression and posterior column reconstruction, posterior spinal shortening osteotomy with direct neural decompression, posterior indirect neural decompression and short-segment spinal fusion combined with vertebroplasty, and so on. Spinal fusion surgery and osteotomy for the treatment of OVF can cause various complications, such as blood loss, infection, and longer hospitalization, which may be magnified in elderly patients. In the treatment of OVF, minimally invasive treatments are required due to the patient's age.

BKP is a minimally invasive surgical treatment and is suitable for OVF in elderly patients. Major complications of BKP for OVF with burst fractures are cement leakage, which may cause remote organ embolism or local chemical or secondary narrowing of the spinal canal by the retropulsion posterior wall in burst fractures. BKP for thoracolumbar burst fracture can achieve a lower rate (9%) of cement leakage, and the longitudinal ligament at the posterior wall also has the potential to prevent cement leakage [4]. In this case, the fracture was an incomplete burst fracture with a partial posterior wall fracture, and therefore, the risk of cement leakage was considered low. Furthermore, careful cement injection under biplane fluoroscopic guidance could further reduce this risk.

Kummell's disease (KD) associated with OVF usually presents with slowly progressive neurological symptoms. For KD, there are some reports of “pivot ligamentotaxis,” such as thoracolumbar burst fracture using a pedicle screw [5]. They achieved a reduction of the fracture fragments and indirectly decompressed the cord with "pivot ligamentotaxis." Anatomically, PLL consists of two layers: the superficial layer (SL) and the deep layer (DL). The SL narrows in the lower lumbar spine. The DL is a fan-like portion and covers about 70% of the horizontal section of the disk. It also covers about 30% of the caudal side of the disk in the vertical direction [6].

In this case, a retropulsed bone fragment with radiculopathy was covered by PLL, and the L3 nerve root was indirectly decompressed by ligamentotaxis induced by fracture reduction using BKP. BKP was less invasive and might be effective in reducing retropulsed bone fragments protruding into the spinal canal. However, if the decompression by ligamentotaxis is insufficient on intraoperative cone beam CT, additional posterior decompression surgery should be considered.

## Conclusions

BKP has proven to be a suitable and effective treatment option for OVF, offering reduced risks of surgical complications. The anatomical structure of PLL plays a significant role in indirect decompression with ligamentotaxis. BKP alone, using the ligamentotaxis of the PLL, is a minimally invasive surgery. However, there is a possibility that the decompression by ligamentotaxis may be insufficient, and if decompression is found to be inadequate on intraoperative cone beam CT, additional posterior decompression surgery should be considered.

## References

[1] Wardlaw D, Cumming Sr, Van Meirhaeghe J (2009). Efficacy and safety of balloon kyphoplasty compared with non-surgical care for vertebral compression fracture (FREE): a randomised controlled trial. Lancet.

[2] Van Meirhaeghe J, Bastian L, Boonen S, Ranstam J, Tillman JB, Wardlaw D (2013). A randomized trial of balloon kyphoplasty and nonsurgical management for treating acute vertebral compression fractures: vertebral body kyphosis correction and surgical parameters. Spine (Phila Pa 1976).

[3] Yaman O, Zileli M, Sharif S (2022). Decompression and fusion surgery for osteoporotic vertebral fractures: WFNS spine committee recommendations. J Neurosurg Sci.

[4] Chung SK, Lee SH, Kim DY, Lee HY (2002). Treatment of lower lumbar radiculopathy caused by osteoporotic compression fracture: the role of vertebroplasty. J Spinal Disord Tech.

[5] Kim HS, Singh R, Adsul NM (2019). Acute burst fracture in Kummell's disease with acute onset neurological deficit: a case report on role of spinal stability and technical notes on "pivot ligamentotaxis". BMC Surg.

[6] Lee SB, Chang JC, Lee GS, Hwang JC, Bae HG, Doh JW (2018). Morphometric study of the lumbar posterior longitudinal ligament. J Korean Neurosurg Soc.

